# Synthesis and Characterization of a Nanocomposite Based on *Opuntia ficus indica* for Efficient Removal of Methylene Blue Dye: Adsorption Kinetics and Optimization by Response Surface Methodology

**DOI:** 10.3390/ijms26146717

**Published:** 2025-07-13

**Authors:** Yasser Boumezough, Gianluca Viscusi, Sihem Arris, Giuliana Gorrasi, Sónia A. C. Carabineiro

**Affiliations:** 1 Laboratory of Environmental Process Engineering, Faculty of Process Engineering, University Salah Boubnider Constantine 3, Constantine 25000, Algeria; yasser.boumezough@univ-constantine3.dz (Y.B.); sihem.arris@univ-constantine3.dz (S.A.); 2 Department of Industrial Engineering, University of Salerno, Via Giovanni Paolo II, 132, 84084 Fisciano, Italy; gviscusi@unisa.it (G.V.); ggorrasi@unisa.it (G.G.); 3 LAQV-REQUIMTE, Department of Chemistry, NOVA School of Science and Technology, Universidade NOVA de Lisboa, 2829-516 Caparica, Portugal

**Keywords:** adsorption, methylene blue, nanocomposite material, water remediation, cactus

## Abstract

In this study, an efficient and cost-effective nanocomposite material based on *Opuntia ficus indica* (cactus) powder modified with iron oxide nanoparticles was developed as an adsorbent for the removal of methylene blue (MB), a common water pollutant. The nanocomposite was synthesized through the co-precipitation method of Fe^2+^ and Fe^3+^ ions and characterized using Fourier-transform infrared spectroscopy (FTIR), scanning electron microscopy (SEM) coupled with energy-dispersive X-ray spectroscopy (EDS) and thermogravimetric analysis (TGA). Batch adsorption experiments were conducted over 24 h, varying different operational conditions, such as pH, temperature and initial pollutant concentration. Furthermore, a Box–Behnken design was employed to develop an empirical model for predicting removal efficiency and optimizing the adsorption conditions. The effects of adsorption variables including contact time (1–60 min), initial MB concentration (20–100 mg/L), pH (2–12), adsorbent dosage (2–6 g/L) and temperature (25–55 °C) on the removal capacity were examined. Under optimal conditions, the maximum removal efficiency of MB reached approximately 96%, with a maximum adsorption capacity of 174 mg/g, as predicted by the Langmuir model. The synthesized cactus/iron oxide nanocomposite demonstrated significant potential as an adsorbent for treating MB-contaminated water.

## 1. Introduction

Environmental degradation caused by anthropogenic activities has impacted ecosystems and public health, particularly in polluted areas. Among various environmental issues, water contamination stands out as one of the most serious, significantly affecting the quality of life [1]. The remediation of water polluted by harmful pollutants is gaining global attention from both basic and applied research communities [2]. According to recent estimates, the textile industry ranks as the fourth-largest sector in resource consumption, after food, housing, and transport, and is one of the most water-polluting industries, contributing to 20% of global water contamination and 10% of global carbon emissions [3].

Hence, the environmental pollution caused by harmful dyes cannot be underestimated. The discharge of untreated dye wastewater, even at low or trace concentrations, poses a growing environmental threat [4], as it significantly alters light refraction and reduces dissolved oxygen levels in water, ultimately leading to the death of aerobic organisms [5]. Therefore, there is an urgent need to address the harmful effects of dye-contaminated wastewater on aquatic ecosystems through the development and application of highly effective and efficient treatment methodologies [6].

Among dye pollutants, methylene blue (MB) is one of the most widely used, with a long history in industrial applications such as textiles, printing and chemical engineering [7]. It is a water-soluble cationic dye with an aromatic structure and poses serious risks to both humans and aquatic organisms. Exposure to MB can lead to adverse health effects, including rapid heartbeat, diarrhea, vomiting, shock, cyanosis, quadriplegia, jaundice and tissue necrosis in humans [8].

Several technologies have been explored for the removal of these types of contaminants, including chemical, physical and biological methods [6]. Biological treatment is a time-consuming process that relies on microbes or enzymes. In contrast, chemical and physical treatments, such as coagulation/flocculation, electrolysis, ozonation, advanced oxidation, direct chemical degradation, adsorption and photocatalysis, are generally faster than biological methods [9,10].

Among the treatment technologies mentioned, adsorption remains the most widely used due to its cost-effectiveness, functional tunability and low energy consumption [11,12]. For these reasons, a variety of adsorbent materials have been employed, including organic clays, zeolites, biochar, activated carbon, metal oxide particles and their composites [13,14,15]. Recently, the selection of appropriate adsorbents has become a critical factor in achieving high adsorption capacity [16].

In recent times, composite nanomaterials with hybrid organic–inorganic components have attracted significant interest due to their potential applications across a wide range of fields [17]. In wastewater treatment, these materials are often engineered to enhance the mechanical stability of adsorbents, as well as to improve their adsorption capacity [18] and separation efficiency [19].

Recently, the application of magnetic nanoparticle technology has attracted significant interest due to its potential to remove heavy metals and organic pollutants from aqueous solutions [20,21]. Furthermore, magnetic separation is a fast and energy-efficient method, unlike conventional techniques such as centrifugation and filtration [22,23]. Among these magnetic adsorbents, Fe_3_O_4_ nanoparticles have gained particular attention due to their exceptional properties, including large surface area, magnetic responsiveness, easy separation and rapid adsorption kinetics [24].

Cactus is a widely cultivated natural product found in arid and desert areas around the world. It is abundant and can be propagated in many different environments globally. Estimates reveal that the exploitation of one hectare of cactus can generate up to 20 tons of dry powder per year [25]. Mexico is considered the only country where cactus cladodes (flat, oval-shaped leaves) are cultivated for commercial purposes, although they remain underutilized in other regions [26]. In addition to their use in food and animal feed in some parts of the world, cactus cladodes have demonstrated effectiveness as eco-friendly materials for wastewater treatment, serving as biosorbents, biocoagulants, and bioflocculants in the pharmaceutical and traditional medicine industries [27,28].

The present paper focuses on the preparation and characterization of a novel cactus/iron oxide hybrid material, representing the first cactus-based iron oxide adsorbent developed for the removal of methylene blue from contaminated water. The efficiency and adsorption potential of the synthesized nanocomposites were thoroughly investigated. Functional groups, elemental composition and morphological characteristics of the prepared nanocomposite were analyzed. Different parameters, including contact time, initial methylene blue concentration, pH, adsorbent dose and temperature, were optimized. Additionally, the regeneration and reusability of the synthesized composite were evaluated. Finally, a statistical approach was employed to predict the system’s response by varying operating conditions such as pH, initial dye concentration and adsorbent dose.

## 2. Results and Discussion

### 2.1. Characterization of the Cactus/Iron Oxide Adsorbent

**Figure 1:**
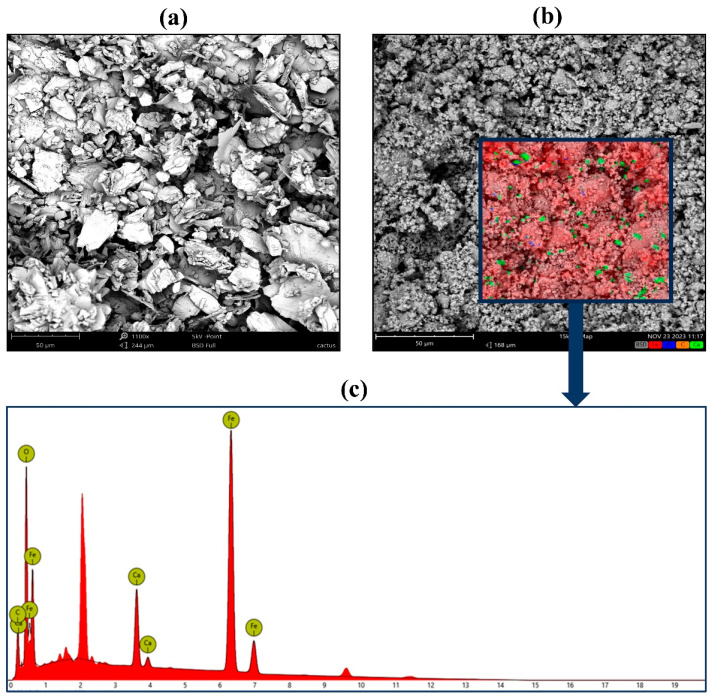
SEM images of (**a**) raw cactus and (**b**) cactus/iron oxide nanocomposite. (**c**) EDX spectrum of the cactus/iron oxide nanomaterial.

SEM analysis was performed to investigate the morphology of the raw cactus before and after magnetization. Figure 1 shows that iron oxide nanoparticles were well dispersed and embedded within the cactus powder. The elemental composition was evaluated using EDX and the results demonstrate the presence of Fe (44.18%), C (29.30%), O (21.13%), and Ca (5.39%) in the cactus/iron oxide composite. The EDX mapping indicates that these elements are evenly distributed on the adsorbent surface, with iron being the predominant element.

As shown in Table 1, the specific surface area of the synthesized material increased compared to the raw cactus powder. The average particle size decreased considerably after synthesis, primarily due to ball milling, which produced a nanoscale material.

**Figure 2:**
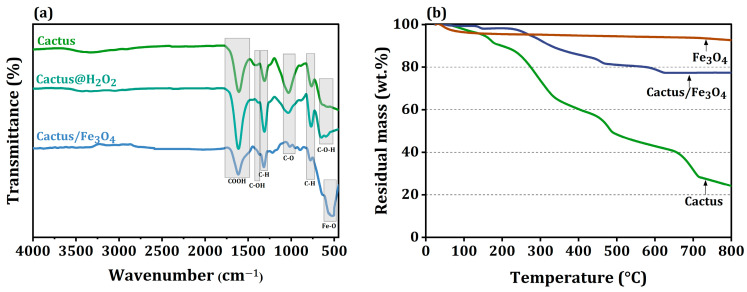
(**a**) FTIR spectra of raw cactus, H_2_O_2_-modified cactus, and cactus/iron oxide nanocomposite material; (**b**) TGA profiles of cactus, iron oxide, and cactus/iron oxide nanocomposite.

FTIR analysis was performed on the raw cactus powder, the modified cactus treated with H_2_O_2_, and the cactus/iron oxide nanocomposite, as shown in Figure 2a.

The absorption band around 1612 cm^−1^ corresponds to the stretching vibrations of carboxylate groups (–COOH) on the surface of the nanocomposite material [29], with higher intensity in the cactus modified with hydrogen peroxide. Additionally, the peaks near 1380 cm^−1^ and 1315 cm^−1^ are attributed to C–OH and C–H deformation, respectively [30,31]. The peak at 1037 cm^−1^ is due to C=O stretching vibrations in lignin in the cactus, although it appears with lower intensity in the modified cactus and the composite [30]. Furthermore, absorption bands observed at 769 cm^−1^ and 637 cm^−1^ can be attributed to C–H bonds and C–O–H groups in the three analyzed materials, respectively [29,30]. The stretching vibration of Fe–O is observed in the wavenumber range from 580 cm^−1^ to 560 cm^−1^, confirming the presence of iron oxide nanoparticles in the composite structure [32].

Figure 2b presents the TGA profile analysis of pure and modified cactus powder. The weight loss from 25 °C to 150 °C is attributed to the evaporation of loosely bound water on the surfaces (5% for cactus and 1.8% for cactus/iron oxide). The degradation process involves multiple weight loss steps in the temperature range of 220–680 °C, which are related to the thermal degradation of cactus, lignin, cellulose, and hemicellulose [33]. In the cactus/iron oxide composite, the removal of components with lower thermal stability, such as pectin and hemicellulose, after treatment with H_2_O_2_, resulted in the disappearance of the characteristic peak around 260 °C, leading to enhanced thermal stability of the fibers. This improvement is likely due to the formation of new lignin–cellulose complexes [34]. The step occurring at approximately 440–460 °C corresponds to the oxidative degradation of the charred residue [35]. The composite did not show significant weight loss with increasing temperature, likely due to the presence of the inorganic component. The residue at 800 °C was 24.2% for cactus and 77.4% for cactus/iron oxide. This improvement is attributed to the higher stability of iron oxide nanoparticles, as well as the ability of inorganic particles to hinder the transmission of oxygen and other gaseous products. This slows the further thermal breakdown of the organic scaffold, creating a barrier between the material and the heat source [35].

### 2.2. Removal of Pollutant (MB) from Water

#### 2.2.1. Effect of Contact Time

**Figure 3:**
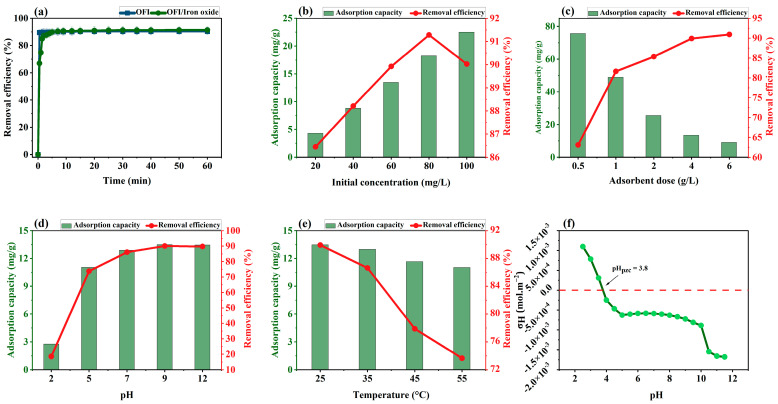
(**a**) Removal efficiency over time for OFI and OFI/iron oxide. Effects of (**b**) initial concentration, (**c**) adsorbent dose, (**d**) pH, (**e**) temperature on OFI/iron oxide, and (**f**) proton surface charge vs. pH for cactus/iron oxide.

Figure 3a shows the effect of contact time on the adsorption efficiency of MB for both raw cactus and the composite. The dye concentration was fixed to 60 mg/L, the adsorbent amount was set to 4 g/L, and the batch studies were conducted at 25 °C and at a pH of 7. Initially, the adsorption rate of the dye was rapid, achieving 90% removal within 5 min. Subsequently, the adsorption efficiency gradually stabilized, reaching equilibrium after 10 min for both the raw cactus and the composite material.

In the initial stage, the adsorbent has a large number of vacant sites on its surface, which explains the rapid adsorption rate of MB molecules interacting with free adsorption sites. As the sorption sites become saturated, the system approaches adsorption–desorption equilibrium due to partial saturation of the binding sites. The improved adsorption capacity after modification can be attributed to the increased surface area of the composite material.

#### 2.2.2. Effect of Initial Concentration of the Solution

The effect of initial concentration on MB adsorption was investigated by varying the solution concentration from 20 mg/L to 100 mg/L. As shown in Figure 3b, the equilibrium adsorption capacity of the adsorbent for MB increased with higher initial dye concentrations. The starting concentration significantly impacted the removal yield of MB, with the adsorption capacity rising from 4.32 mg/g at C_0_ = 20 mg/L to 22.5 mg/g at C_0_ = 100 mg/L.

The dye removal was concentration-dependent, as higher initial concentrations increased the driving force, which helped overcome the resistance to mass transfer between the aqueous and solid phases [36].

#### 2.2.3. Effect of Adsorbent Amount

The amount of adsorbent nanocomposite is crucial for cost-effectiveness and environmental sustainability in the adsorption process, as it directly impacts the feasibility and efficiency of wastewater treatment [2]. In this study, the adsorption of 60 mg/L MB at pH 7 was evaluated using adsorbent doses of 0.5, 1, 2, 4, and 6 g/L, as shown in Figure 3c. The results indicated that the adsorption efficiency for doses of 4 and 6 g/L were nearly identical, with 4 g/L identified as the optimal adsorbent dose for MB removal.

As the adsorbent amount increases, more adsorption sites become available, leading to higher MB removal efficiency. However, the adsorption capacity, which is related to the mass of the adsorbent, decreases as the dose increases from 0.5 to 6 g/L. Excessive adsorbent dosages can lead to particle agglomeration, reducing the specific surface area and limiting the exposure of adsorption sites, ultimately decreasing adsorption efficiency.

#### 2.2.4. Effect of Initial Solution pH

The pH of the aqueous medium significantly impacts dye adsorption by altering the properties of either the adsorbent surface or the adsorbate. The effect of pH was studied by varying the initial dye solution pH from 2 to 12 at 25 °C, with results displayed in Figure 3d. The removal efficiency of MB increased from 18.6% to 90% as the pH rose from 2 to 12, while the adsorption capacity improved from 2.98 to 13.46 mg/g.

As shown in Figure 3d, when the pH exceeded the point of zero charge (pH_PZC_), the adsorbent surface became deprotonated and negatively charged. This enhanced the electrostatic attraction between the cationic MB molecules and the negatively charged surface [36]. Consequently, lower adsorption of MB onto the iron oxide/cactus composite was observed at acidic pH values. This behavior is further explained by the reduced competition between cationic MB and hydrogen ions for adsorption sites as the pH increases.

#### 2.2.5. Effect of Temperature

The effect of temperature on adsorption was studied at four different temperature values: 25 °C, 35 °C, 45 °C and 55 °C, as shown in Figure 3f. The adsorption conditions were as follows: an initial dye concentration of 60 mg/L, a solution pH of 7 and an adsorbent dose of 4 g/L. As shown in Figure 3e, the adsorption process was negatively affected by the increase in temperature. To further investigate this observation, a thermodynamic analysis was conducted.

### 2.3. Adsorption Isotherms

Numerous adsorption isotherm models can be employed to evaluate the interaction between the liquid phase (adsorbate solution) and the solid phase (adsorbent). These models assess key parameters, such as the maximum adsorption capacity, adsorption strength and the affinity of the adsorbate molecules for the adsorbent surface [37]. The Langmuir isotherm model, which describes homogeneous monolayer adsorption, is commonly used to evaluate the maximum adsorption capacity and the Langmuir constant, *K_L_* (L mg^−1^), which indicates the affinity of the adsorption sites for the adsorbate [38]. Based on the separation factor (*R_L_*), the adsorption process can be classified as positive (0 < *R_L_* < 1), negative (*R_L_* > 1), linear (*R_L_* = 1) and irreversible (*R_L_* = 0). The linear regression of the Langmuir isotherm equation is expressed by Equation (1):

(1) $$\frac{C_{e}}{q_{e}}=\frac{1}{b{\;q}_{max}}+\frac{C_{e}}{q_{max}}$$

In this equation, *q_e_* (mg/g) and *q_max_* (mg/g) represent the equilibrium adsorption capacity and the maximum adsorption capacity, respectively. The constant *b* (L/mg) is the Langmuir adsorption constant, while *C_e_* (mg/L) is the equilibrium concentration of the adsorbate.

The Freundlich model describes a heterogeneous multilayer adsorption system. The linear form of the Freundlich isotherm is given by Equation (2):

(2) $$\log q_{e}=\log K_{F}+\frac{1}{n}\log C_{e}$$

where *K_F_* (L/mg) represents the adsorptive capacity and 1/*n* indicates the adsorption intensity, which is related to the relative distribution of energy and the heterogeneity of the adsorbent sites.

The Temkin isotherm model is useful for explaining the adsorption potentials of the adsorbent for removing adsorbate molecules. The linear form of the Temkin isotherm is given by Equation (3):

(3) $$q_{e}=B_{j}\times \ln K_{t}+B_{j}\times lnC_{e}$$

where *K_t_* (L/g) is the equilibrium binding constant and *B_j_* (J/mol) is the heat of adsorption.

**Figure 4:**
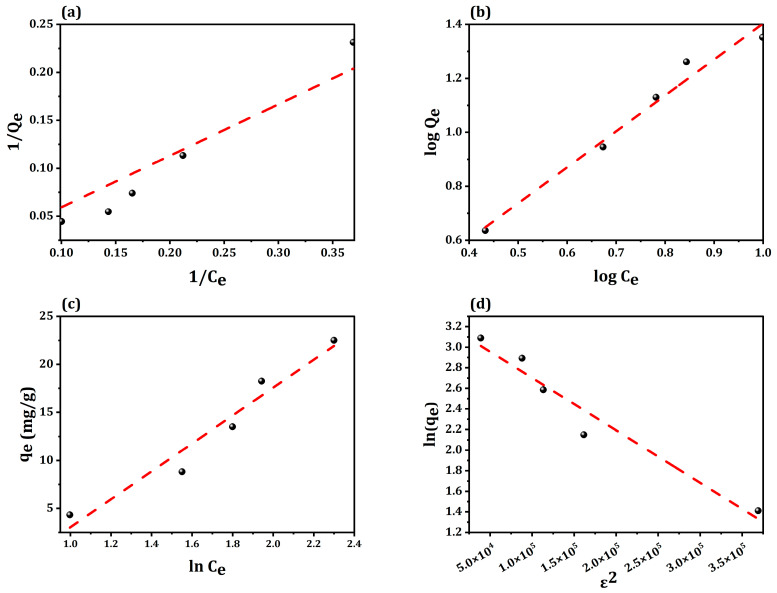
Linear plots of (**a**) Langmuir, (**b**) Freundlich, (**c**) Temkin and (**d**) Dubinin–Radushkevich adsorption isotherm models.

Dubinin–Radushkevich (D-R) isotherm is used to differentiate between physical and chemical adsorption based on the mean free energy, as given by Equation (5). The linear regression form of the D-R model is represented by Equation (4):

(4) $$\ln q_{e}=\ln q_{m}-\beta \varepsilon^{2}$$

(5) $$E=\frac{1}{\surd 2\beta }$$

where *β* (mol^2^/kJ^2^) is the D-R constant and *E* (kJ/mol) is the mean free energy. Figure 4 displays the Langmuir, Freundlich, Temkin and D-R isotherm fittings for the adsorption of MB onto cactus/iron oxide. The corresponding parameters for the adsorption isotherms are provided in Table 2.

The figures and *R*^2^ values from Table 2 indicate that the adsorption data were well-fitted with both Langmuir and Freundlich isotherms. The Langmuir isotherm fitting suggests that the cactus/iron oxide composites have homogeneous adsorbent surfaces involving a monolayer adsorption process with similar adsorption sites. In contrast, the Freundlich isotherm indicates that the surfaces are covered with multilayers of adsorbate.

Additionally, the *R_L_* value suggests that the adsorption process is linear. Since the average free energy exceeds 8 kJ/mol, the D-R model indicates that the process is primarily chemisorption.

Equilibrium isotherm parameters and their corresponding values are reported in Table 2.

### 2.4. Analysis of Thermodynamic Parameters

The free energy of adsorption for MB (*ΔG*°) can be evaluated using Equation (6):

(6) $$\Delta G{}^{\circ}=-RT\times ln(K_{L})$$

where *K_L_*, defined as *q_eq_*/*c_eq_*, represents the adsorption affinity. Enthalpy (*ΔH*°) and entropy (*ΔS*°) were estimated using Equation (7) by extrapolating the slope and the intercept of the plot [39].

(7) ΔG°=ΔH°−TΔS°

**Figure 5:**
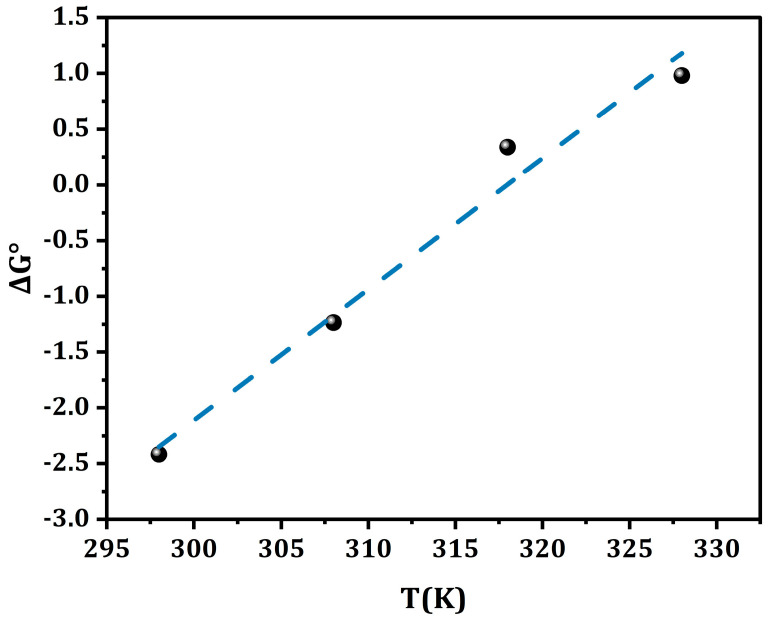
Gibbs’s free energy trend as a function of temperature.

The graph of *ΔG*° vs. temperature (*T*), shown in Figure 5, provides further insight into the thermodynamic parameters. The slope of the plot yields *ΔS*°, and the intercept gives *ΔH*°. From the evaluation of the slope and intercept, the values of *ΔS*° and *ΔH*° were found to be −0.118 kJ/mol K and −37.61 kJ/mol respectively.

As depicted in Table 3, the designed adsorbents exhibited a *ΔH*° < 0, indicating that the reaction was exothermic and that a decrease in temperature favored the adsorption process. A *ΔS*° < 0 suggests that the adsorption process was characterized by a decrease in entropy, reflecting a reduction in randomness at the interface between MB and cactus/iron oxide composite [40].

### 2.5. Analysis of Adsorption Kinetics

Adsorption behaviors were investigated using the pseudo-first-order (PFO) model (Equation (8)), the pseudo-second-order (PSO) model (Equation (10)), the intraparticle diffusion kinetics (IPD) model (Equation (9)), and the Elovich model (Equation (10)). The corresponding results are presented in Table 4.

(8) $$\ln\left(q_{e}-q_{t}\right)=lnq_{e}-k_{1}t$$

(9) $$\frac{t}{q_{t}}=\frac{t}{q_{e}}+\frac{1}{k_{2}q_{e}^{2}}$$

(10) $$q_{t}=k_{d}t^{\frac{1}{2}}+C$$

where *q_t_* and *q_e_* (mg/g) represent the amount of dye adsorbed at time *t* and at equilibrium, respectively; *k*_1_ (min^−1^) is the rate constant for the PFO model and *k*_2_ (g/(mg·min)) is the rate constant for the PSO kinetic model, *C* is a constant related to the boundary layer thickness; and *k_d_* (g/(mg·min^1/2^)) is the rate constant for the IPD model, *K_e_* is the adsorption rate constant for the Elovich equation, and *A* is a constant.

**Figure 6:**
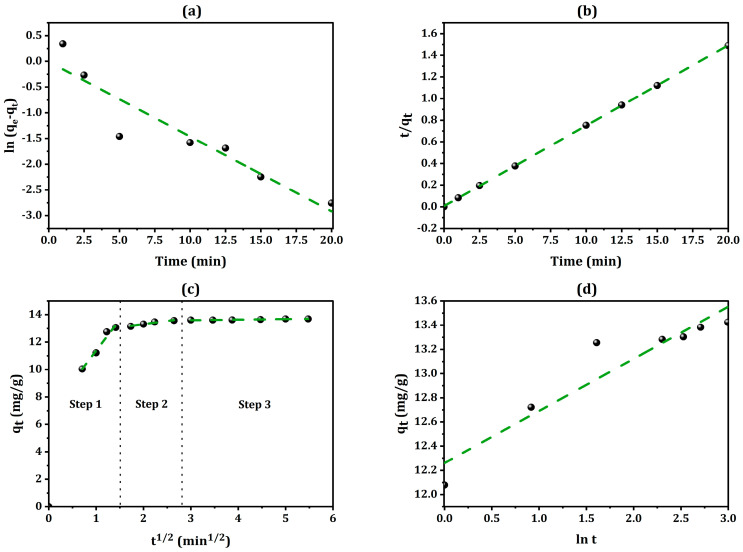
Linear plotting of (**a**) pseudo-first-order, (**b**) pseudo-second-order, (**c**) intraparticle diffusion, and (**d**) Elovich adsorption kinetic models.

The comparison of kinetic model parameters based on their *R*^2^ values, as shown in Table 4 and Figure 6, reveals that the adsorption behavior of MB on cactus/iron oxide is best explained by the PSO kinetic model. This is supported by its high (*R*^2^ > 0.999) and excellent goodness-of-fit (RMSE ≤ 1 × 10^−4^). Additionally, the *Q_2_* value calculated by the PSO model was close to the experimental (*Qe*,*exp*) value for MB at different temperatures.

The PSO model suggests that the adsorption process likely involves chemical adsorption and provides a comprehensive interpretation of the process, including liquid film diffusion, intraparticle diffusion, and surface adsorption. This also implies that the rate of adsorption does not depend solely on the dye concentration but rather on the adsorption capacity of the composite material [41].

The fitting results from the IPD model, shown in Figure 6c, reveal three distinct stages, corresponding to different diffusion mechanisms. The slopes of these stages (*K_id_*_1_ > *K_id_*_2_ > *K_id_*_3_) indicate that the adsorption process is initially rapid on the external surface due to the availability of active sites. In the second stage, MB molecules diffuse into the particle pores, increasing the adsorption capacity. Finally, in the third stage, the adsorption capacity stabilizes as the adsorbent gradually approaches equilibrium.

Non-linear regression of the adsorption models was compared with results from linear regression. Interestingly, both approaches produced very similar outcomes, with no significant differences in the estimated parameter values.

To further validate this, model comparisons were conducted using the F-test, Akaike Information Criterion (AIC), and Bayesian Information Criterion (BIC), all of which indicated no notable discrepancies or underestimation in the fitted parameters. Based on these results, the linear regression approach was retained, as it sufficiently captures the adsorption behavior observed in this study.

### 2.6. Boyd Model

The Boyd equation can be used to understand the rate-controlling step of the adsorption process, as given by Equation (11):

(11) $$F=1-\frac{6}{\pi^{2}}\times {exp}^{-Bt}$$

where *F* is the fraction of adsorbed solute (*q_t_*) relative to the equilibrium capacity (*q_e_*). The *B_t_* values can be evaluated using Equation (12):

(12) $$B_{t}=-0.4977-ln(1-F)$$

**Figure 7:**
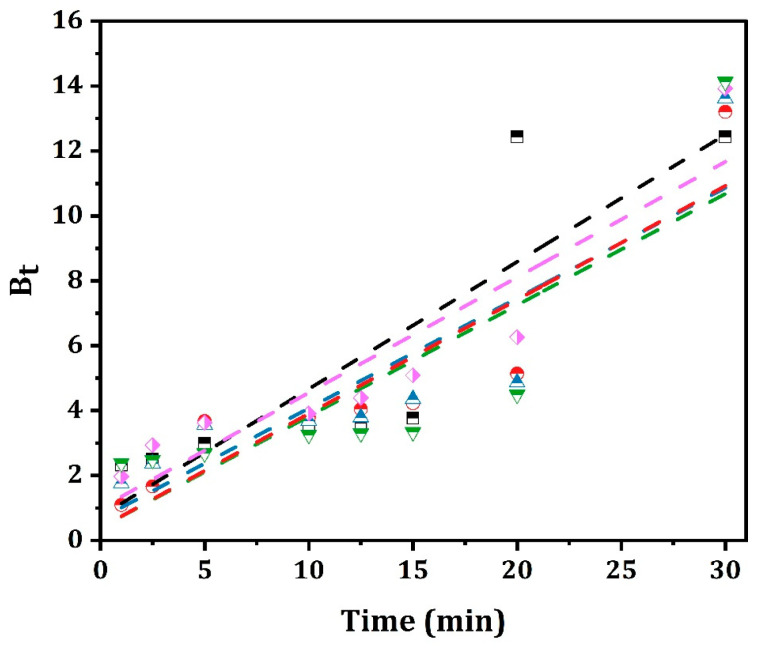
Boyd’s plot.

The Boyd plot, which represents *B_t_* against time *t*, can help determine the rate-controlling step of the adsorption (Figure 7). If the plot produces a straight line passing through the origin, it suggests that pore diffusion is the limiting step. On the other hand, if the plot does not pass through the origin, film diffusion or external mass transport is likely the controlling factor [42].

The *R*^2^ values for Boyd’s equation ranged from 0.66 to 0.83. Additionally, the curves did not pass through the origin, indicating that the adsorption of MB dye is not controlled by pore diffusion.

### 2.7. Adsorption Mechanism

**Figure 8:**
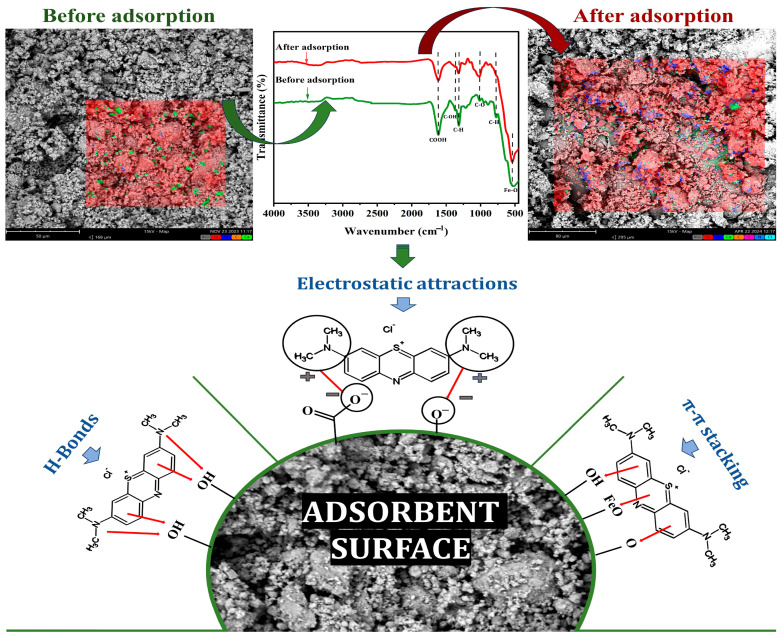
Proposed mechanism for MB removal.

To gain a deeper understanding of the adsorption process and the mechanisms involved in the removal of MB by the cactus/iron oxide nanocomposite, FTIR analysis was performed both before and after the adsorption process, as depicted in Figure 8.

The proposed mechanism aims to simplify the understanding of the complex adsorption process. Figure 8 suggests that the adsorption of MB on the cactus/iron oxide nanocomposite is primarily driven by electrostatic attractions. Specifically, the positively charged cationic dye interacts with the negatively charged hydroxyl (–OH) groups on the surface of the cactus [43]. These functional groups act as active sites for binding MB molecules onto the adsorbent surface.

The involvement of these functional groups is supported by a decrease in the intensity of the OH stretching band in the 3200–3600 cm^−1^ range, the C=O carbonyl stretching at 1728 cm^−1^, and the C=O band at 1110 cm^−1^, all of which suggest that the –OH and C=O groups play a key role in the adsorption process. Additionally, the amino groups in the MB dye molecules form dipole–dipole hydrogen bonds with the –OH and –COOH groups on the cactus surface [44]. Furthermore, the benzene rings of the MB molecules may interact with the hydrogen atoms on the cactus surface through Yoshida H-bonds. The iron oxide nanoparticles, containing numerous vacant O^2−^ sites around Fe^2+^ and Fe^3+^ ions, also contribute to the adsorption by interacting with the positive charges of the MB dye [45].

Lastly, n-π stacking is another interaction that aids the adsorption process. This interaction occurs between the electron-donating surface of the adsorbent and the electron-accepting π bonds in the MB dye molecule.

### 2.8. Optimization by Box Behnken Design

**Figure 9:**
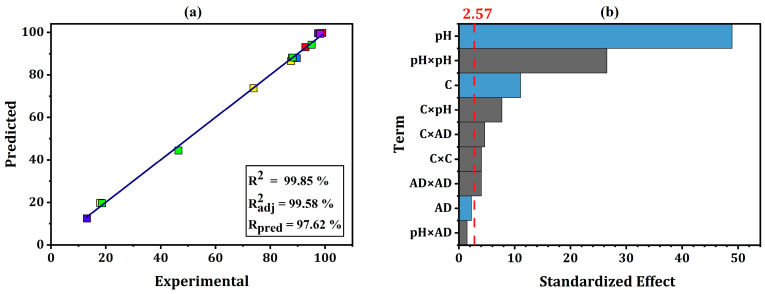
(**a**) Scatter plot of predicted vs. experimental MB removal; (**b**) Pareto chart of the standardized effects of pH, initial concentration, and adsorbent dose.

To investigate the effect of the studied factors and their interactions on the removal process, adsorption experiments were conducted based on the experimental runs generated by the RSM-BBD model. As depicted in Figure 9a, the results were validated using the regression method, which yielded a high correlation coefficient (*R*^2^ > 0.99).

(13) $$\begin{array}{ll} R\left(\%\right)=-40.54 & -0.085X_{1}+26.051X_{2}+5.33X_{3}+0.002714X_{1}X_{1}-1.1375X_{2}X_{2}\\ & -1.082X_{3}X_{3}-0.03978X_{1}X_{2}+0.0597X_{1}X_{3}-0.154X_{2}X_{3} \end{array}$$

The impact of the factors and their interactions on the adsorption process can be quantified using the coded equation of the model. As shown in Figure 9b, the Pareto chart reveals that pH is the most influential factor governing the process. The initial concentration also significantly impacts adsorption, while the adsorbent dose is considered an insignificant factor, according to the model. All interactions are considered influential, except for the interaction between pH and adsorbent dose.

**Figure 10:**
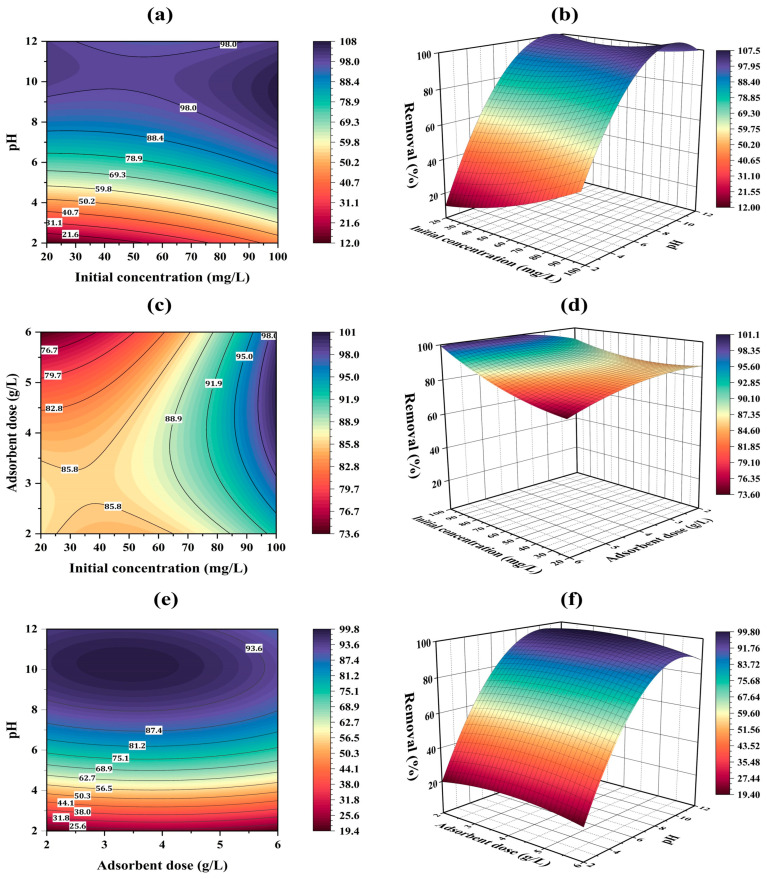
(**a**) Contour plot and (**b**) 3D plots showing the interactive effect of initial concentration and pH; (**c**) Contour plot and (**d**) 3D plots showing the interactive effect of initial concentration and adsorbent dose; (**e**) Contour plot and (**f**) 3D plots the interactive effect of pH and adsorbent dose.

Figure 10 illustrates the interactive effects of each pair of parameters on dye removal efficiency, while keeping other parameters constant. The curvature of the contours and three-dimensional plots provides a detailed insight into the analyzed factors and their interactions.

As shown in Figure 10a,b, pH is the most influential factor, with a positive correlation to the removal percentage of MB dye. The initial concentration has a relatively minor effect compared to pH, indicating that the removal efficiency increases in basic pH solutions. Figure 10c,d display the effects of adsorbent dose and initial concentration on removal efficiency. The nearly horizontal surfaces suggest an insignificant interaction between these two factors. At high initial concentrations and adsorbent doses, a maximum removal rate is achieved, indicating that higher initial concentrations require a sufficient amount of adsorbent.

Similarly, Figure 10e,f demonstrate that removal efficiency has a positive correlation with pH, while the effect of adsorbent dose is minimal compared to pH. This further confirms that pH is the key factor influencing the adsorption of MB by the magnetic nanocomposite material.

The predicted model estimates a high removal yield at an initial concentration of 20 mg/L, a pH of 10.98 and an adsorbent amount of 2.24 g/L, with a significant desirability score of 1, confirming the model’s accuracy. To validate these optimization results, three experiments were carried out under these conditions, yielding a very high removal rate of 96.1%.

### 2.9. Regeneration and Reusability Potential

**Figure 11:**
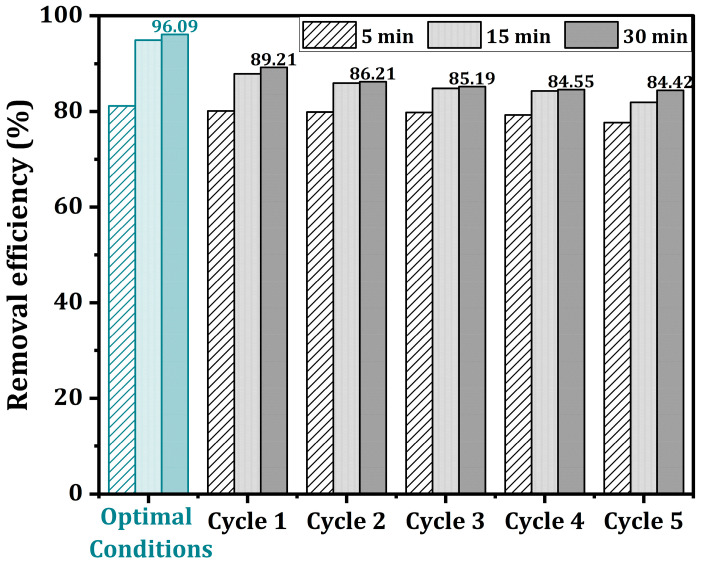
Reusability study of cactus/iron oxide.

As shown in Figure 11, after 5 consecutive adsorption–desorption cycles, the color removal efficiency decreased by less than 5%. This reduction can be attributed to the saturation of active sites and a decrease in the surface area. The slight decrease in adsorption efficiency (approximately 0.8%) between the 3rd and the 5th cycles, may be due to some dye molecules not being fully desorbed. This result confirms that the cactus/iron oxide nanocomposite remains effective and exhibits excellent regeneration stability, even after multiple cycles.

### 2.10. Comparison of the Current Material with Other Nanocomposite Materials for the Removal of Organic Pollutants

The combination of magnetic materials and biomaterials is widely regarded as an economic, sustainable and environment-friendly approach, significantly contributing to the remediation of contaminated waters. To minimize production costs, it is essential that biomaterials are used cyclically and remain in their natural state [46].

To evaluate the performance and adsorption capacity of our composite material, a comparison was made with other magnetic composites, as shown in Table 5. Our cactus/iron oxide nanocomposite demonstrates better performance than that of magnetic aerogel and sulfobetaine-modified magnetic nanoparticles but has slightly lower performance than that of magnetic activated charcoal.

However, our cactus/iron oxide nanocomposite offers several advantages over traditional magnetic activated charcoal. Firstly, it is derived from sustainable and renewable cactus materials, making it more environmentally friendly. The synthesis process is cost-effective and less energy-intensive, which contributes to lower production costs and reduces environmental impact. Additionally, the presence of the iron oxide component enables easy separation from the solution, simplifying the post-treatment process. These factors, along with potential benefits in selectivity and biocompatibility, position the cactus/iron oxide nanocomposite as a competitive and sustainable alternative for adsorption applications.

## 3. Materials and Methods

### 3.1. Materials

The *Opuntia ficus indica* cactus cladodes were collected from the Oued Athmania region in eastern Algeria. The chemicals used in the synthesis and adsorption processes were of analytical grade and purchased from Sigma-Aldrich (St. Louis, MO, USA): Methylene blue (CAS 122965-43-9), iron sulfate heptahydrate (CAS 7782-63-0), iron chloride hexahydrate (CAS 10025-77-1), hydrogen peroxide solution (35%) (CAS 7722-84-1) and ammonium hydroxide solution (30–33%) (CAS 1336-21-6). These chemicals were used without further purification in the preparation of the cactus/iron oxide nanocomposite and for subsequent adsorption experiments.

#### Preparation of the Magnetic Nanocomposite Material

The cactus/iron oxide nanocomposite adsorbent was prepared in three steps as described below.

First, mature cactus cladodes were washed with distilled water to remove any small particles, impurities and spikes. The cleaned cladodes were then dried in an oven at 80 °C for 48 h to remove any residual moisture. Once dried, the cladodes were crushed using a grinder and sieved to obtain a particle size of <100 µm.

After that, the cactus powder was chemically modified with H_2_O_2_ (1 M). A total of 10 g of cactus powder was added to 200 mL of a 1 M H_2_O_2_ solution. The mixture was agitated at 400 rpm for 4 h at 80 °C. After this treatment, the mixture was filtered, and the solid material was washed with distilled water until neutral pH of 7 was reached.

The cactus powder was then incorporated into the synthesis of the cactus/iron oxide nanocomposite using the co-precipitation method. Accordingly, 1 g of the modified cactus powder was added to an aqueous solution containing Fe^3+^ and Fe^2+^ ions in a 2:1 molar ratio (400 mL). The mixture was agitated for 2 h at 80 °C. Ammonium hydroxide (NH_4_OH) solution was then added dropwise until the pH of the mixture reached 9. The mixture was kept under agitation at 400 rpm for 24 h to ensure complete reaction.

After the reaction, the material was separated from the solution using a magnet, confirming the magnetic properties of the composite. The composite was then washed until a neutral pH was achieved and dried at 80 °C for 12 h.

**Scheme 1:**
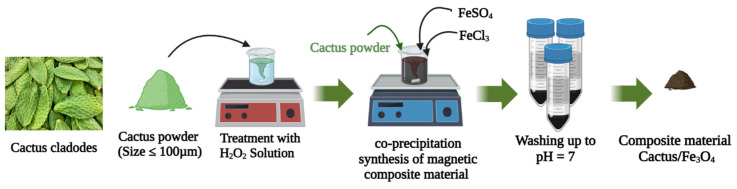
Preparation and synthesis of the nanocomposite material.

The dried nanocomposite powder was further processed by placing it in a planetary ball mill, which was operated at 300 rpm for 10 min to achieve finer particle size and better dispersion of the iron oxide nanoparticles within the cactus matrix. The entire preparation process is summarized in Scheme 1.

### 3.2. Methods

#### 3.2.1. Characterization

The structural features of the cactus/iron oxide nanocomposite were analyzed using Fourier Transform Infrared Spectroscopy (FTIR). FTIR spectra were recorded using a Bruker spectrometer (Bruker Daltonics GmbH & Co. KG, Bremen, Germany).

Scanning Electron Microscopy combined with Energy Dispersive X-ray Spectroscopy (SEM-EDX) was used to visualize and determine the morphological features and elemental distribution within the nanocomposite. This analysis was performed using a Phenom ProX microscope (Phenom-World BV, Eindhoven, The Netherlands), which operates in high-vacuum mode. The EDX detector integrated with the SEM allowed for elemental analysis to confirm the presence of iron (Fe) and other constituent elements in the nanocomposite.

The thermal stability of the cactus/iron oxide nanocomposite was evaluated using a thermogravimetric analyzer (Mettler TC-10 thermobalance from Mettler-Toledo GmbH, Greifensee, Switzerland). TGA measurements were carried out over a temperature range of 30 °C to 700 °C at a heating rate of 10 °C/min to determine the decomposition patterns and stability of the material.

The mean dimensions of the nanocomposite particles and the zeta potential of both the raw and modified cactus were measured using Dynamic Light Scattering (DLS). A Zetasizer Nano S (Worcestershire, UK) was used to measure the particle size distribution and assess the surface charge. These measurements were conducted in triplicate to ensure reproducibility.

The specific surface area (S_BET_) was determined by N_2_ adsorption using a Costech Sorptometer 1042 analyzer at −196 °C (Costech Microanalytical, Tallinn, Estonia). Prior to analysis, the samples were degassed at 150 °C for 30 min to remove any adsorbed moisture or volatile materials. The Brunauer-Emmett-Teller (BET) method was used to calculate the surface area.

The point of zero charge (pH_PZC_) of the cactus/iron oxide nanocomposite was determined using a titration method. An aliquot of NaCl solution (0.1 M, 40 mL) containing 200 mg of the nanocomposite was equilibrated for 30 min. NaOH was added dropwise to adjust the pH to a constant value, and titration was performed with HCl solution (1 M) to lower the pH to approximately 2.5. The proton surface charge density, *σ_H_* (mol·m^−2^), was then calculated using Equation (14):

(14) $$\sigma_{H}=\frac{V}{m\times S}\times \left\{\left({\left[H^{+}\right]}_{b}-{\left[H^{+}\right]}_{s}\right)-\left(\frac{K_{w}}{{\left[H^{+}\right]}_{b}}-\frac{K_{w}}{{\left[H^{+}\right]}_{s}}\right)\right\}$$

where *V* is the volume (0.04 L); *S* the specific surface area; *m* the sample mass; [*H*^+^] represents the proton concentration in the solution (M); *K_w_* the dissociation constant of water. The intercept of *σ_H_* on the x-axis was used to evaluate the pH corresponding to the pH_PZC_, which helps in understanding the surface characteristics of the adsorbent and its interaction with various ions in the solution.

To consider the presence of ions, ionic strength was considered, and activity values were calculated through Debye–Hückel theory. The subscripts b and s refer to blank and sample solutions, respectively. The intercept of *σ_H_* on the x-axis allows the evaluation of the pH corresponding to the point of zero charge PZC.

The value 1 × 10^−14^ for *K_w_* is valid under conditions of infinite dilution at 25 °C. However, in the presence of significant ionic strength, such as that introduced by NaCl (0.1 M), NaOH and HCl in the present study, the ionic strength can influence the effective value of *K_w_*. To address this, we recalculated the proton surface charge density (*σ_H_*) by incorporating the effect of ionic strength. The molar ionic strength (I) was calculated using the equation:

$$I=\frac{1}{2}\sum_{i=1}^{n}C_{i}\times {Z_{i}}^{2}$$

where *C*_*i*_ represents the concentration of each ion and *Z*_*i*_ is the charge of each ion.

Additionally, we applied the Debye–Hückel equation to estimate the activity coefficients of the ions, allowing for a more accurate determination of the apparent value of *K_w_* under the given ionic conditions.

Although the material was not characterized using vibrating sample magnetometry (VSM) or Mössbauer spectroscopy, it exhibited strong magnetic behavior. During synthesis, the magnetic fraction was separated using a magnet, and magnetic separation was consistently employed throughout the experiments.

#### 3.2.2. Batch Adsorption Experiments

Batch adsorption experiments were carried out in 20 mL vials containing the MB solution and the synthesized magnetic cactus/iron oxide nanocomposite. The vials were agitated at a speed of 400 rpm to ensure uniform mixing. The amount of adsorbent used was 4 g/L and the experiments were conducted at ambient temperature.

The adsorption process was investigated by varying the following parameters: contact time (1–60 min), MB initial concentration (20–100 mg/L), adsorbent amount (0.5–6 g/L), pH (2–12), and temperature (25–55 °C). All experiments were performed in triplicate to ensure the reliability and reproducibility of the results.

After the adsorption process, the final MB concentration in the solution was measured using an ultraviolet–visible (UV-vis) spectrophotometer (Jenway 6715 from fisher scientific, Waltham, MA, USA) by recording the absorbance at a wavelength of λ = 664 nm, which corresponds to the maximum absorbance of MB. The removal efficiency (*R*, %) and uptake capacity (*Q*, mg/g) were calculated using the following Equations (15) and (16):

(15) $$R(\%)=\frac{C_{0}-C_{e}}{C_{0}}\times 100$$

(16) $$Q=\frac{(C_{0}-C_{e})\times V}{m}$$

where *C*_0_ and *C_e_* (mg/L) represent the initial and equilibrium concentration of the MB solution, respectively; *V* (L) is the volume of the MB solution; *m* (g) is the amount of adsorbent.

#### 3.2.3. Optimization by Response Surface Methodology

Response surface methodology (RSM) is a powerful approach for statistical modeling of a process, aiming to establish an approximate relationship between a set of independent parameters and a given response [28]. RSM utilizes multivariate mathematical and statistical techniques based on a series of planned experiments to determine the optimal response of the parameters under study. The Box Behnken design (BBD) is widely used in process optimization to minimize the number of required experiments while maximizing the information gained about the relationship between the factors and the studied response [24,25]. In this case, the operating conditions and their interactions were examined using BBD with three levels and three factors. The ranges and levels of the selected factors are detailed in Table 6.

#### 3.2.4. Regeneration Experiments

The reusability study of the nanocomposite material was conducted under optimal conditions over five adsorption–desorption cycles. After each adsorption cycle, the composite was treated with 50% *v*/*v* ethanol. The desorbed adsorbent was then washed three times with distilled water to ensure complete removal of the MB dye from its surface, after which it was dried at 80 °C for 6 h before being reused.

## 4. Conclusions

The nanocomposite material based on modified cactus was successfully synthesized using a simple and cost-effective method. The structure and morphology of the material were characterized using various techniques, including FTIR, TGA, SEM-EDX, and N_2_ adsorption. The characterization data revealed the formation of a highly porous composite with non-uniform incorporation of iron oxide nanoparticles.

The application of the synthesized cactus/iron oxide nanoparticle composite as an adsorbent was studied for the removal of MB dye. The effects of contact time and pH demonstrated rapid adsorption, reaching about 90% removal in 10 min, with enhanced MB adsorption at basic pH. This is due to the negative charges present on the adsorbent surface, which create electrostatic interactions with the cationic MB molecules.

Analysis of adsorption isotherms, kinetics, and thermodynamics described the cactus/iron oxide composite adsorption of MB as spontaneous, feasible, exothermic chemisorption with a maximum adsorption capacity of 174 mg/g according to Langmuir isotherm. An empirical model obtained using RSM confirmed the significant effect of pH on MB remediation. Optimization revealed that a maximum removal efficiency of 96.1% was achieved at pH = 10.98, with an adsorbent dose of 2.24 g/L of adsorbent dose and an initial concentration of 20 mg/L. Model validation showed a strong correlation between experimental and predicted values.

Hence, this paper demonstrates the potential of a new iron oxide composite prepared from natural adsorbents, which exhibits excellent performance in removing toxic cationic dyes from water.

## Figures and Tables

**Table 1:** Parameters of cactus and cactus/iron oxide samples.

Sample	Specific Surface Area (m^2^/g)	Mean Dimension (nm)	Zeta Potential (mV)
Cactus	3.3	11,000	−21 ± 0.21
Cactus/iron oxide	39.3	57.7	−27 ± 0.33

**Table 2:** Isotherm parameters for MB removal.

Isotherm Model	Parameters	Values
Langmuir	*q_m_* (mg/g)	174.82
	*K_L_* (L/mg)	0.011
	*R_L_*	1
	*R* ^2^	0.971
Freundlich	*K_f_* (L/mg)	1.18
	1/*n*	1.33
	*R* ^2^	0.975
Temkin	*B_j_*	14.53
	*K_T_* (L/mg)	0.45
	*R* ^2^	0.948
Dubinin–Radushkevich	*q_m_* (mg/g)	24.8098
	*ß* (mol^2^/kJ^2^)	5.09 × 10^−6^
	*E* (kJ/mol)	3,133,035
	*R* ^2^	0.9505

**Table 3:** Thermodynamic parameters for MB removal.

Temperature (K)	*ΔG*° (kJ/mol)	*ΔS*° (kJ/mol K)	*ΔH*° (kJ/mol)
298	−2.42	−0.118	−37.61
308	−1.24		
318	0.34		
328	0.97		

**Table 4:** Kinetic parameters for MB removal.

Kinetic Model	Parameters	Values
Pseudo-first-order	*K*_1_ (min^−1^)	−0.007
	*q_e_* (mg·g^−1^)	0.991
	*R* ^2^	0.8796
Pseudo-second-order	*K*_2_ ((g/mg)min)	0.815
	*q_e_*, cal(mg/g^−1^)	13.454
	*R* ^2^	0.9999
Intraparticle diffusion Step 1	*K_id_* ((mg/g)min^−0.5^)	4.539
	*C* (mg·g^−1^)	6.836
	*R* ^2^	0.9683
Intraparticle diffusion Step 2	*K_id_* ((mg/g)min^−0.5^)	0.465
	*C* (mg·g^−1^)	12.375
	*R* ^2^	0.9478
Intraparticle diffusion Step 3	*K_id_* ((mg/g)min^−0.5^)	0.040
	*C* (mg·g^−1^)	13.473
	*R* ^2^	0.9448
Elovich	* α *	2.322
	* β *	99.17 × 10^10^
	*R* ^2^	0.898

**Table 5:** Comparison of organic pollution adsorption on different magnetic composite and nanocomposite materials.

Adsorbent	Pollutant	Conditions	*Q_max_* (mg·g^−1^)	References
Geopolymer/Fe_3_O_4_	Acid green 16	Equilibrium time 30 min pH 2.3 Adsorbent dosage 0.75 g·L^−1^	400	[47]
Chitosan-polypyrrole-Fe_3_O_4_	Acid green	Equilibrium time 60 min pH 5.4 Adsorbent dosage 1 g·L^−1^	26.2	[48]
Magnetic hydrochar/chitosan	Malachite green	Equilibrium time 420 min pH 7.5 Adsorbent dosage 0.4 g·L^−1^	420.02	[49]
Mag-γ-Fe_2_O_3_-cross linked-chitosan composite	Methyl orange	Equilibrium time 100–270 min pH 4 Adsorbent dosage 1 g·L^−1^	29.50	[50]
Fe_3_O_4_/corn stalks biochar	Crystal violet	Equilibrium time 5 min pH 6 Adsorbent dosage 0.25 g·L^−1^	349.4	[51]
Sulfobetaine-modified magnetic nanoparticles	Methyl blue	Equilibrium time 15 min pH 2–2.5 Adsorbent dosage 10 mg	127.06	[52]
Magnetic black carbon	Carbamazepine	Equilibrium time 3 min pH 2–10 Adsorbent dosage 0.5 g·L^−1^	31.8	[53]
Polypyrrole/GO/biochar	Carbamazepine	Equilibrium time 90 min pH 7.8 Adsorbent dosage 1.4 g·L^−1^	45.04	[54]
Carbon nanotube/polyaniline/magnetite	Methylene blue	Equilibrium time 180 min pH 2.94–10.03 Adsorbent dosage 1.5 g·L^−1^	166.66	[55]
Magnetic activated charcoal	Methylene blue	Equilibrium time 60 min pH 9.6 Adsorbent dosage 1.46 g·L^−1^	455	[56]
Magnetic aerogel	Methylene blue	Equilibrium time 6 h Natural pH	83.6	[57]
Cactus/iron oxide nanocomposite	Methylene blue	Equilibrium time 10 min pH 10.98 Adsorbent dosage 2.24 g·L^−1^	174.82	This work

**Table 6:** Parameter intervals used in the Box Behnken design.

Factors	Symbol	Coded and Real Values		
		−1	0	+1
Initial concentration (mg/L)	X_1_	20	60	100
pH	X_2_	2	7	12
Adsorbent dose (g/L)	X_3_	2	4	6

## Data Availability

The datasets used and/or analyzed during the current study are available from the corresponding author upon reasonable request.
